# Temporal trends in arthropod abundances after the transition to organic farming in paddy fields

**DOI:** 10.1371/journal.pone.0190946

**Published:** 2018-01-11

**Authors:** Masaru H. Tsutsui, Kazuhiko Kobayashi, Tadashi Miyashita

**Affiliations:** 1 Laboratory of Biodiversity Science, Graduate School of Agricultural and Life Sciences, The University of Tokyo, Tokyo, Japan; 2 Laboratory of Sustainable Agriculture, Graduate School of Agricultural and Life Sciences, The University of Tokyo, Tokyo, Japan; Charles University, CZECH REPUBLIC

## Abstract

Organic farming aims to reduce the effect on the ecosystem and enhance biodiversity in agricultural areas, but the long-term effectiveness of its application is unclear. Assessments have rarely included various taxonomic groups with different ecological and economic roles. In paddy fields with different numbers of years elapsed since the transition from conventional to organic farming, we investigated changes in the abundance of insect pests, generalist predators, and species of conservation concern. The abundance of various arthropods exhibited diverse trends with respect to years elapsed since the transition to organic farming. Larval lepidopterans, *Tetragnatha* spiders, and some planthoppers and stink bugs showed non-linear increases over time, eventually reaching saturation, such as the abundance increasing for several years and then becoming stable after 10 years. This pattern can be explained by the effects of residual pesticides, the lag time of soil mineralization, and dispersal limitation. A damselfly (*Ischnura asiatica*) did not show a particular trend over time, probably due to its rapid immigration from source habitats. Unexpectedly, both planthoppers and some leafhoppers exhibited gradual decreases over time. As their abundances were negatively related to the abundance of *Tetragnatha* spiders, increased predation by natural enemies might gradually decrease these insect populations. These results suggest that the consideration of time-dependent responses of organisms is essential for the evaluation of the costs and benefits of organic farming, and such evaluations could provide a basis for guidelines regarding the length of time for organic farming to restore biodiversity or the economic subsidy needed to compensate for pest damage.

## Introduction

Agricultural landscapes are characterized by high spatial heterogeneity and intermittent human disturbances, and they have been maintained by traditional management for over 2000 years [1,2]. During this period, many organisms are believed to have adapted to the variable environments, resulting in rich biodiversity in the human-altered landscapes. However, recent modernization or intensification of farmland management has led to the severe decline of many taxonomic groups worldwide, including insects, amphibians, and birds [3,4,5]. Environmental problems other than biodiversity loss, such as food safety and soil deterioration, have also become major issues in agricultural modernization [6,7,8].

To address problems related to agricultural modernization, organic farming and wildlife-friendly (WF) farming are carried out [9]. Organic farming uses neither pesticides nor chemical fertilizers, while WF farming uses smaller quantities compared to conventional farming. Many studies have evaluated the effectiveness of organic/WF farming on biodiversity, but the results are inconsistent and rather mixed [2,10], including positive, neutral, and even negative effects. There are several explanations for these differences, including differences in productivity determined by regional climates [11,12] and landscape structures surrounding the crop fields [4,13]. Another explanation that has received recent attention is variation in the time elapsed since the conversion to organic/WF farming; the responses of biodiversity to changes in farming practices may not be immediate, but may exhibit time lags [10,14,15]. Assessing this time lag appears to be crucial for evaluating the minimum duration of organic/WF farming necessary to restore biodiversity and thereby can facilitate agro-environmental policymaking [2,16].

Rice fields occupy about 11% of the world’s agricultural lands [17] and about 90% are distributed in Asia [18]. With the emergence of significant environmental issues associated with agricultural modernization or intensification, organic/WF farming has increased since the late 1990s [16]. As rice paddies are important habitats for wetland-dependent species, the transition to organic/WF farming is expected to restore high levels of biodiversity [19,20]. Since organic and WF farming may also increase pest insects, it is important to identify how pest insects, their natural enemies, as well as organisms of conservation concern change in abundance in response to the conversion to organic/WF farming. Nevertheless, no studies, to our knowledge, have clarified the effect of the time since the transition to organic/WF farming on organisms in rice paddy fields.

In this study, we examined the abundance of foliage-dwelling arthropods in rice fields under organic farming for various numbers of years after the conversion from conventional practices. Our target taxa include various arthropods with different functions, covering insect pests (lepidopterans, leafhoppers, planthoppers, and stinkbugs), natural enemies (*Tetragnatha* spiders), and damselflies (which are occasionally used as an indicator of biodiversity conservation). The purpose of this study was two-fold: (1) to evaluate the effectiveness of organic farming on various functional groups of arthropods by comparing conventional and organic paddy fields, and (2) to evaluate the effect of the time since the transition to organic farming on these arthropods, which is the main objective. We expected most species groups to exhibit a delayed positive response to the initiation of organic farming because soil properties important for sustaining biodiversity in crop fields, such as the mineralization rate of nitrogen, generally respond slowly [10,21,22], and the dispersal capacity of some organisms could also constrain immediate responses [10,23]. In particular, *Tetragnatha* spiders are expected to show a more delayed response compared with other groups owing to their higher trophic positions in food webs than those of other arthropods.

## Materials and methods

### Study sites

Field studies were located in Nogi (36°14′N 139°44′E; elevation: 25 m), Tochigi Prefecture, eastern Japan. This area is characterized by a paddy-dominated landscape mixed with settlements and scattered secondary forests. A farmer initiated the organic management of paddy fields in 1992 and has gradually expanded the management strategy to other fields. His fields had therefore been under organic management for various numbers of years from zero (the first year) to more than 20 years at the time of this study. Organic paddy fields at 3 years, 4 years, 10 years, and 18 years after the transition to organic farming were examined. In total, 12 organic fields were selected, so that each year-group had triplicate paddies. In addition, four paddy fields under conventional management in the proximity of the organic fields were selected. All of the study fields were located within a 1 km^2^ area, at an average distance (±SD) of 288 ± 160 m. The average area (±SD) of each paddy field was 2153 (±1795) m^2^ and did not differ significantly between organic and conventional fields. Most of the habitats next to the focal paddy fields were other paddy fields, with the exception that 1 of 4 edges of five paddy fields were adjacent to abandoned fields. The land owner, Hiroyuki Tateno, gave permission to conduct the field survey.

Under organic farming, paddy fields were left fallow after harvest in autumn until the next spring, allowing vigorous growth of winter weeds, consisting mainly of a graminaceous species, *Alopecurus aequalis*. The fields were ploughed and paddled a few times in early- to mid-June, not only to plant rice, but also to incorporate the weeds into the soil to provide organic matter. Neither chemical fertilizers nor pesticides were applied; only rice bran and guano were applied to soils when rice seedlings were transplanted. Herbicides were not used, but summer weeds that compete with rice were effectively controlled in most fields. More details of this organic farming strategy can be found in [24]. In conventional farms, in contrast, chemical fertilizers, pesticides, as well as herbicides were used (S1 Table).

### Sampling of arthropods

Insects and spiders were surveyed three times in the rice season, i.e., late July, early August, and late August, in 2015. These periods include late vegetative to early grain-filling stages of rice plants and are also the periods when insect and spider abundances are relatively high.

Arthropods were captured from the upper vegetation layer of rice plants using an insect net with a 100-cm rod (42 cm in diameter). Sweeping was conducted at eight points in each paddy field, covering an area of 3.14 m^2^ around each point, at the edge (from the paddy levee to 1 m) of each paddy field. Samples captured by sweeping were preserved in 80% ethanol and were identified to the species level when possible using a stereomicroscope. We focused on six taxa, i.e., lepidopterans (*Naranga aenescens* and *Parnara guttata*), leafhoppers, planthoppers, stink bugs, *Tetragnatha* spiders, and damselflies (*Ischnura asiatica*). These taxa can be categorized into four functional groups consisting of insect pests, natural enemies, and species of conservation concern. *N*. *aenescens* and *P*. *guttata* are leaf-eating lepidopterans that sporadically damage rice plants, and only larvae were captured. Leafhoppers and planthoppers are sap feeders, each consisting of several species (S1 Appendix, S1 File), and are known to be vectors of rice diseases. Stink bugs also consist of several species (S1 Appendix, S1 File); they damage rice grains by piercing their stylets (mouth parts) in the early heading stage. *Tetragnatha* spiders are dominant natural enemies in paddy fields [25], and are regarded as a biodiversity indicator in paddy fields [26]. *Ischnura asiatica* is a common damselfly inhabiting lowland ponds and wetlands, and is used as an indicator of habitat restoration in human-dominated landscapes [27,28].

### Statistical analysis

A two-way ANOVA was used to test the effects of farming types (organic vs. conventional) and seasons (late July, early August, and late August) on the abundance of each taxon. Here, the total number of individuals captured at all points in a paddy field was regarded as the abundance and used for statistical analyses. Prior to analysis, abundance data were square-root transformed to meet normality assumptions. Although a generalized linear model with a Poisson or negative binomial error term would be better for this analysis, the results are not affected by the model (S2 Table).

To determine how taxon abundance changes with the time since the transition to organic farming, four models were fitted in an exploratory manner to the year-abundance relationship, i.e., Michaelis-Menten saturation, power law, linear, and intercept-only null models. Notice that only data from organic paddy fields were used here. The model performance was evaluated based on AIC (Akaike information criterion). Competing models with ΔAIC (i.e., the difference in AIC values) <2 exhibit similar performance [29]. Here, ΔAIC is the difference in AICs between the null and best models. If ΔAIC was larger than 2, the best model was regarded as meaningful, and the fitted curve was drawn; otherwise, no trends in abundance were found. Since model selection was employed here, no significance testing was performed.

Unlike other taxa, both planthoppers and leafhoppers in early August exhibited an unexpected negative trend over time (see Results). Thus, we tested whether this trend was related to the abundance of spiders, which are potential natural enemies. A simple regression model with a Poisson or negative binomial error term was applied to this analysis for all pest insects (planthoppers, leafhoppers, stink bugs, and lepidopterans). Notice that the spider abundance in late July was used here, as the predation effects on prey populations generally have a lag time.

All statistical analyses were performed using R v3.2.2 [30].

## Results

### Organic vs. conventional farming

The two-way ANOVA revealed differences among farming types in arthropod abundances. For hemipterans (stink bugs, plant hoppers, and leafhoppers), there were no consistent effects across seasons (Table 1). However, a significant interactive effect of farming type and season was found for planthoppers and leafhoppers; they were more abundant in conventional faming plots in early-August, but less abundant or equally abundant in late-August (S1 Appendix).

**Table 1:** Results of two-way ANOVAs showing the effects of farming type and season on the abundance of various arthropods (*: p<0.05; **: p<0.01; ****:p<0.001).

Variable	DF	MS	*F*	*P*	
Stink bug					
Season	2	6.97	4.37	0.019	*
Farming type	1	1.40	0.88	0.354	
Interaction	2	2.52	1.58	0.218	
Planthopper					
Season	2	417.00	46.29	<0.001	***
Farming type	1	1.90	0.21	0.650	
Interaction	2	39.80	4.42	0.018	*
Leafhopper					
Season	2	413.30	25.66	<0.001	***
Farming type	1	34.60	2.15	0.150	
Interaction	2	74.40	4.62	0.015	*
Lepidopteran					
Season	2	9.62	4.60	0.016	*
Farming type	1	43.70	20.87	<0.001	***
Interaction	2	10.02	4.79	0.013	*
*Tetragnatha* spider					
Season	2	16.90	9.87	<0.001	***
Farming type	1	19.07	11.13	0.002	**
Interaction	2	1.55	0.91	0.411	
Damselfly					
Season	2	0.53	0.54	0.590	
Farming type	1	7.70	7.78	0.008	**
Interaction	2	0.10	0.10	0.903	

In contrast to the above pest insects, there was a highly significant effect of farming type on the abundances of larval lepidopterans (Table 1), with higher abundances for organic farming (S1 Appendix). There was a significant interactive effect of farming type and season for larval lepidopterans, with a much stronger positive effect of organic farming in August.

There was also a highly significant positive effect of organic farming on the abundances of *Tetragnatha* spiders (Table 1, S1 Appendix), although no interactive effect was found. The same pattern was found for damselflies, with a higher abundance in organic farming areas across seasons (Table 1, S1 Appendix).

### Effect of the time since the transition to organic farming

**Fig 1:**
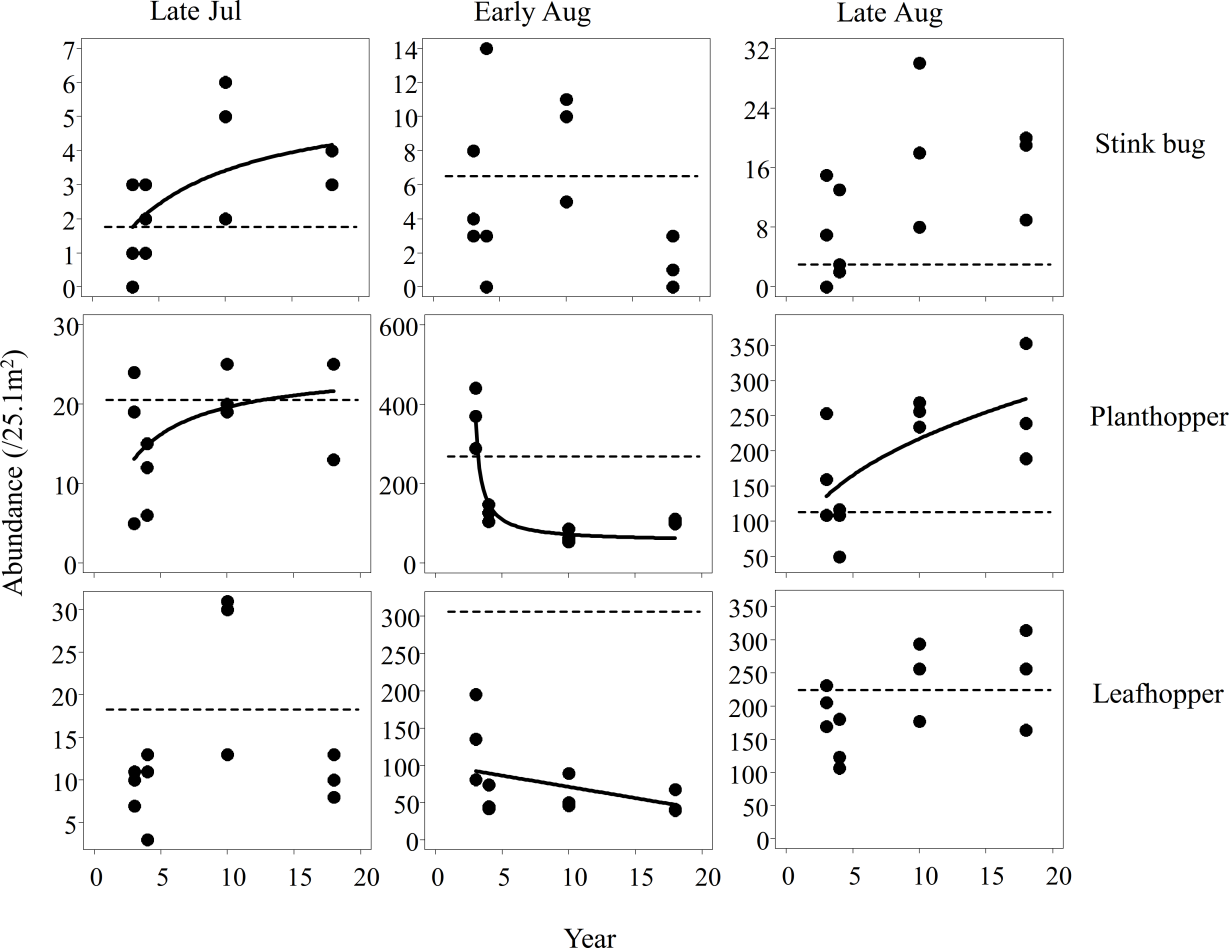
Relationships between the time since the transition to organic farming and the abundance of organisms (stink bug, planthopper, and leafhopper species). Regression curves were drawn when the best model had ΔAIC > 2 in comparison to the null model. Horizontal dashed lines indicate the mean values in control fields subjected to conventional farming.

**Fig 2:**
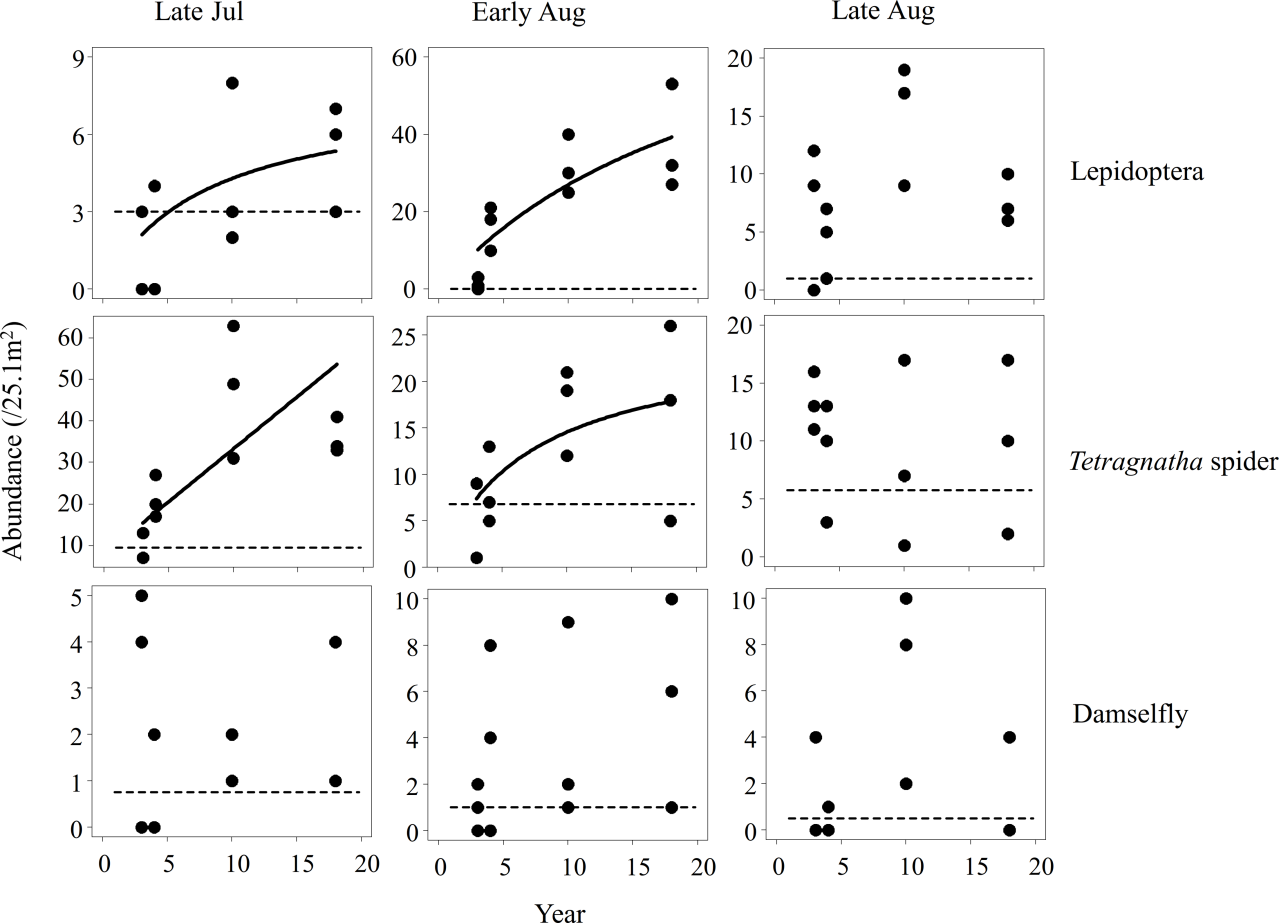
Relationships between the time since the transition to organic farming and the abundance of organisms (Lepidoptera, *Tetragnatha* spider, and damselfly species). Regression curves were drawn when the best model had ΔAIC > 2 in comparison to the null model. Horizontal dashed lines indicate the mean values in control fields subjected to conventional farming.

All arthropods, except for damselflies, showed trends in abundance with respect to years since the transition to organic farming at least once in the three seasons (Figs 1 and 2). Seven out of nine graphs in Figs 1 and 2 show gradual increases in abundance with years elapsed, while the remaining two cases showed decreasing trends.

Stink bugs showed an increasing trend in the early season (late July), but no trends in early and late August, when their abundance became high (S1 Appendix). Planthoppers showed variable trends, i.e., increasing trends in the early and late seasons, but a decreasing trend in the middle season (early August) (Fig 1). Leafhoppers exhibited a similar decreasing trend in the middle season (Fig 1). The decreasing pattern was nonlinear for planthoppers and linear for leafhoppers (Table 2).

**Table 2:** Regression models relating years since transition to organic farming (X) to abundance of arthropods (Y). Three different models (Michaelis-Menten, power law, linear) were applied, and the best model was chosen based on AIC. Here, ΔAIC is the difference of AICs between null and the best models. If ΔAIC was larger than 2, the best model was regarded as meaningful, and the fitted curve was drawn in Figs 1 and 2.

Arthropod group	Season	Baet model	ΔAIC	Formula
Stink bug	Late Jul	Michaelis-Menten	4.05	Y = 5.75X / (X+6.82)
	Early Aug	Linear-	0.61	
	Late Aug	Linear-	1.09	
Planthopper	Late Jul	Michaelis-Menten	2.86	Y = 24.87X / (X+2.69)
	Early Aug	Michaelis-Menten	17.6	Y = 53.72X / (X-2.56)
	Late Aug	Michaelis-Menten	5.42	Y = 347.71X / (X+5.22)
Leafhopper	Late Jul	Null*	0	
	Early Aug	Linea	2.15	Y = -3.05X+101.80
	Late Aug	Power-law*	1.58	
Lepidoptera	Late Jul	Michaelis-Menten	2.21	Y = 7.74X / (X+7.99)
	Early Aug	Michaelis-Menten	10.35	Y = 91.05X / (X+23.72)
	Late Aug	Null-	0	
*Tetragnatha* spider	Late Jul	Linear	7.06	Y = 2.55X+7.80
	Early Aug	Michaelis-Menten	3	Y = 24.86X / (X+7.08)
	Late Aug	Power-law-	0.16	
Damselfly	Late Jul	Null-	0	
	Early Aug	Null-	0	
	Late Aug	Null-	0	

Lepidopterans showed increasing trends with years since the transition in the early and middle seasons, but no trend in the late season (Fig 2). The increasing pattern was non-linear in the two seasons, reaching saturation (Fig 2, Table 2).

*Tetragnatha* spiders also increased as the time since the transition to organic farming increased in the two earlier seasons, when they were abundant, but this pattern disappeared in the late season, when they became less abundant (Fig 2). The increase over time was linear in late July and non-linear in early August (Table 2).

For damselflies, there was no trend with respect to the time since the transition to organic farming, and the null model always exhibited the lowest AIC value across seasons (Fig 2).

### Relationship between spiders and pest insects

**Fig 3:**
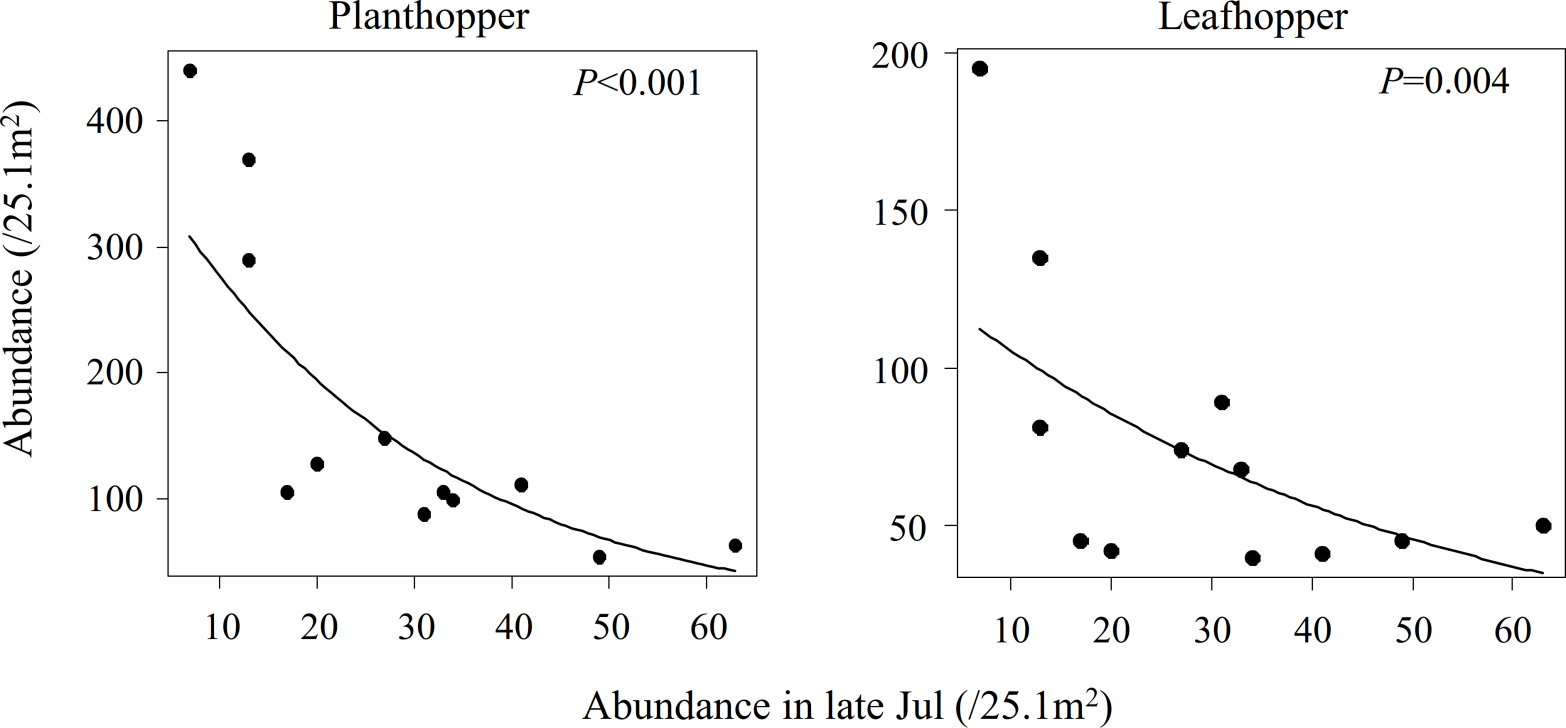
Relationship between the spider abundance in late July and insect abundance in early August.

The abundances of both planthoppers and leafhoppers in early August had a negative relationship with the abundance of *Tetragnatha* spiders in late July (Fig 3). This relationship was due to the temporal shift in the relative abundances of predator and prey species, i.e., from a state of low-spider and high-hopper abundances to high-spider and low-hopper abundances. However, a similar negative relationship was not found for stinkbugs (*z* = 0.527, *P* = 0.598) and lepidopterans (*z* = 4.712, *P* < 0.001); notice that lepidopteran abundance exhibited a positive association with spider abundance. Additionally, the abundance relationship between spiders and planthoppers or leafhoppers became positive in late August (Planthopper, *z* = 2.418, *P* = 0.016; Leafhopper, *z* = 2.175, *P =* 0.030), suggesting the disappearance of top-down effects in this season.

## Discussion

Our results demonstrated that the time since the transition from conventional to organic farming appeared to influence the abundance of some arthropods in paddy fields. Moreover, the shape of the time-abundance relationship occasionally exhibited a non-linear saturation pattern, i.e., abundance increased for several years, but did not appreciably increase from 10 years onward. This suggests that a non-equilibrium state of arthropod populations could last for a decade after the transition to organic farming. To our knowledge, this study provides the first evidence for time-dependent responses of organism in rice fields. Earlier studies on cereal fields in Europe have also shown transient abundance or richness dynamics for plants [31], butterflies [14,15], and microbes [32] in response to changes in farming practices. Most of these studies have shown a continual increase for several years or more, with the exception of weeds and weed-dependent moths, which show a peak in abundance/richness after a few years [23]. The results of our study are therefore in agreement, in part, with earlier studies of cereal croplands. We expected *Tetragnatha* spiders to show a more delayed response to organic farming due to their higher trophic position, but such a pattern was not evident in comparison to other arthropods.

Several factors are thought to explain the delayed or gradual response of organisms, including dispersal limitation (or “colonization credit”; [33]), effects of residual pesticides and/or chemical fertilizer in soil [15,34], and lag time to the mineralization of organic matter [10,22]. In the present study, some taxa showed a gradual increase since the transition to organic farming, i.e., larval lepidopterans, *Tetragnatha* spiders, and some planthoppers and stink bugs. Among the above factors, dispersal limitation may not be the main cause of the gradual increase, as individual paddy fields were the unit to which organic farming was applied, so immigration into such fields from adjacent habitats is likely to occur in a short period. Nevertheless, dispersal limitation might explain the observed changes in *Tetragnatha* spiders, to some degree. These spiders are known to aggregate in patches with high prey availability [35], but their mechanism of movement basically involves changing their web sites day-by-day, probably in a trial-error manner. This process appears to be slow in comparison to the movement of freely flying insects.

The effect of residual pesticides is a likely cause for delayed responses because some pesticides applied in our conventional paddy fields have residual toxicity [36]. In particular, fipronil residues remain in paddy soils for over a year, and decrease not only target insect pests (lepidopterans and hemipterans), but also other insects, including chironomid larvae [37]. This could further affect the abundance of *Tetragnatha* spiders, which feed mainly on adult chironomids in paddy fields [26,38], resulting in a delayed response.

The gradual increase in soil nutrient availability after the transition to organic farming due to elevated microbial decomposition [22,39] may also have increased herbivorous insects via enhanced rice plant quality. In our paddy fields, soil nitrogen availability was actually higher in a field under organic farming for 12 years than in a field undergoing organic farming for 2 years [24]. Such a bottom-up effect could further cascade up to spiders via an increased prey abundance because chironomids, major prey of *Tetragnatha* spiders, are known to increase with increasing nutrient availability in aquatic sediments [40,41]. More research is needed to clarify the relative importance of these factors (dispersal limitation, residual pesticides, and nutrient availability) for determining the delayed responses of arthropod abundance.

Unexpectedly, both planthoppers and leafhoppers exhibited a gradual decrease, not an increase, in abundance with the time the since transition to organic farming. This detrimental effect on these insect pests, although beneficial to farmers, cannot be explained by any of the mechanisms mentioned above. It is possible that enhanced predation on these insects by natural enemies, an indirect effect of organic farming, mediated top-down population control. Actually, there was a negative relationship between the abundance of *Tetragnatha* spiders and the abundance of both planthoppers and leafhoppers. As late July was the season when spiders were most abundant, this might have suppressed populations of planthoppers and leafhoppers in early August. It is noteworthy that a negative relationship was not detected in late August, when the spider abundance decreased in paddy fields. *Tetragnatha* spiders are dominant predators in paddy ecosystems, and are suspected to control insect pest populations; they eat leafhoppers and stink bugs, as determined by direct observations and DNA analyses of stomach contents [42, 43]. Nevertheless, *Tetragnatha* spiders alone do not appear to have such strong top-down effects. We consider that *Tetragnatha* abundance only represents the abundance of natural enemy assemblages that were not examined in this study.

The rice insect pests currently causing major economic losses in Japan are stink bugs, which pierce immature rice grans, resulting in damaged “pecky rice” [44]. Our results showed that organic farming did not induce a consistently higher abundance of stink bugs across seasons. This is surprising because conventional farming used insecticides targeting stink bugs (S1 Table). There was also no trend in abundance after the transition to organic farming in late August, when stink bugs were most abundant. Stink bugs causing spotted rice damage are known to inhabit abandoned paddy fields, levees, and road verges in earlier seasons, from which they migrate to paddy fields during the heading stage of rice plants [45]. Therefore, migration from such alternative habitats may have masked the effects of pesticides in conventional farms. Actually, there were densely vegetated patches of Poaceae in our study sites, with a large number of stink bugs. Mowing management of these alternative habitats in this season could help reduce the impacts of stink bugs under organic farming.

We used the damselfly *Ischnura asiatica* as a proxy for species of conservation concern; the species responded positively to organic farming, with no lag. The immediate response is probably due to immigration from source habitats, such as water-holding abandoned paddy fields, as this species could travel around 1 km in the adult stage [27]. Although small organic/WF farms are not likely to become substitutes for source habitats, they could function to link such habitats and to offer refuges when source habitats are disturbed and degraded. Furthermore, since traditionally managed paddy fields sometimes harbor endangered insects, including damselflies, diving beetles, and aquatic bugs [46,47,48], well-connected organic farms in wider areas could restore these populations, especially in complex landscapes with a larger species pool [4,49].

## Conclusions

We found appreciable effects of the time since the transition to organic farming on organismal abundance in paddy fields, but the response types varied among taxa, with increasing, steady, and even decreasing patterns. The time-abundance relationship occasionally exhibited a non-linear saturation pattern, and a transient non-equilibrium state could last for a decade. Moreover, differences in responses might be partially explained by predator-prey interactions. Although our results are not conclusive owing to the limited number of replications and the time-for-space substitution approach, they strongly suggest that consideration of the time-dependent responses of organisms is essential for the evaluation of the costs and benefits of organic/WF farming. Such evaluations could facilitate the establishment of guidelines regarding how long organic/WF farming should be continued to restore biodiversity or the economic subsidy needed to compensate for pest damage.

## Supporting information

S1 Table Types of fertilizer and pesticides that were applied to conventional paddy fields in our study area. (PDF)

S2 Table Results of GLM showing the effects of farming type and season on the abundance of various arthropods. (PDF)

S1 Appendix Mean (±SE) abundance of various arthropods in different seasons. Left and right bars indicate organic and conventional farming, respectively.
(PDF)

S1 File Arthropod abundance data used for the analyses. (XLSX)
