# Supplementary material for: Temporal trends in arthropod abundances after the transition to organic farming in paddy fields
Source: PLoS One. 2018 Jan 11;13(1):e0190946. doi: 10.1371/journal.pone.0190946 (PMC5764318; doi:10.1371/journal.pone.0190946)
Supplement: S1 Table — (PDF) [file pone.0190946.s001.pdf]

S1 Table. Types of fertilizer and pesticides that were applied to conventional paddy fields in our study area.

Type	Season	Chemical component	Formulation
Fertilizer	Late April	phosphoric acid, nitrogen, potassium	Granular
Insecticide (nursery box)*	Early May	carbosulfan	Granular
		clothianidin	Granular
		fipronil, isoprothiolane	Granular
Herbicide*	Late May - early June	esprocarb, bensulfuron-methyl	Granular
		chyhalojop butil, dimethametryn, halosulfuron-methyl	Granular
		imazosulfuron, cafenstrole, benzobicyclon	Wettable powder, granular
Insecticide (field)	Late July	etofenprox, azoxystrobin	Wettable powder

*One of the chemical component items was applied to a given paddy field
