# Supplementary material for: Temporal trends in arthropod abundances after the transition to organic farming in paddy fields
Source: PLoS One. 2018 Jan 11;13(1):e0190946. doi: 10.1371/journal.pone.0190946 (PMC5764318; doi:10.1371/journal.pone.0190946)
Supplement: S2 Table — (PDF) [file pone.0190946.s002.pdf]

S2 Table. Results of GLM showing the effects of farming type and season on the abundance of various arthropods.

	Early Aug	Late Aug	Farming type	EA×Farming	LA×Farming
Stink bug	0.015 *	<0.001 ***	0.138		
Planthopper	<0.001 ***	<0.001 ***	0.582	0.458	0.086 ▪
Leafhopper	<0.001 ***	<0.001 ***	0.433	0.048 *	0.676
Lepidoptera	0.052 ▪	0.262	0.767	<0.001 ***	0.039 *
Tetragnatha spider	0.001 **	<0.001 ***	0.001 **		
Damselfly	0.212	0.605	0.007 **		

P<0.1: ▪, *P*<0.05:*, *P*<0.01**, *P*<0.001: ***
