# Supplementary material for: Temporal trends in arthropod abundances after the transition to organic farming in paddy fields
Source: PLoS One. 2018 Jan 11;13(1):e0190946. doi: 10.1371/journal.pone.0190946 (PMC5764318; doi:10.1371/journal.pone.0190946)
Supplement: S1 Appendix — Left and right bars indicate organic and conventional farming, respectively. (PDF) [file pone.0190946.s003.pdf]

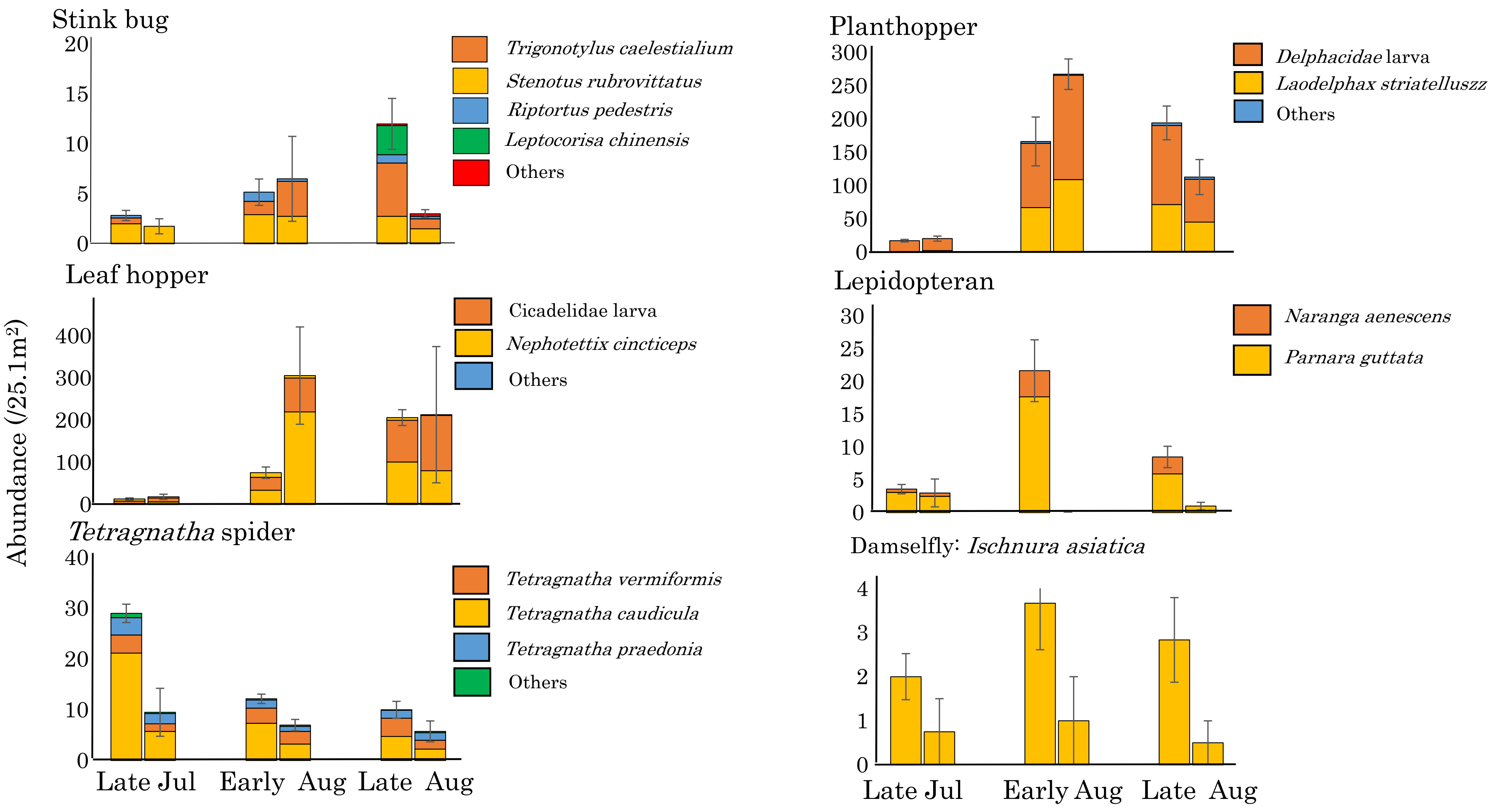

S1 Appendix. Mean (±SE) abundance of various arthropods in different seasons. Left and right bars indicate organic and conventional farming, respectively.
